# Preoperative risk factors and cumulative incidence of temporary ileostomy non-closure after sphincter-preserving surgery for rectal cancer: a meta-analysis

**DOI:** 10.1186/s12957-024-03363-z

**Published:** 2024-04-12

**Authors:** Fan He, Chenglin Tang, Fuyu Yang, Defei Chen, Junjie Xiong, Yu Zou, Dongqin Zhao, Kun Qian

**Affiliations:** Department of Gastrointestinal Surgery, The First Affiliated Hospital of Chongqing Medical University, Chongqing, 400016 China

**Keywords:** Temporary ileostomy, Risk factors, Rectal cancer, Meta-analysis

## Abstract

**Background:**

Temporary ileostomy (TI) has proven effective in reducing the severity of anastomotic leakage after rectal cancer surgery; however, some ileostomies fail to reverse over time, leading to conversion into a permanent stoma (PS). In this study, we aimed to investigate the preoperative risk factors and cumulative incidence of TI non-closure after sphincter-preserving surgery for rectal cancer.

**Materials and methods:**

We conducted a meta-analysis after searching the Embase, Web of Science, PubMed, and MEDLINE databases from their inception until November 2023. We collected all published studies on the risk factors related to TI non-closure after sphincter-preserving surgery for rectal cancer.

**Results:**

A total of 1610 studies were retrieved, and 13 studies were included for meta-analysis, comprising 3026 patients. The results of the meta-analysis showed that the identified risk factors included older age (*p* = 0.03), especially > 65 years of age (*p* = 0.03), male sex (*p* = 0.009), American Society of Anesthesiologists score ≥ 3 (*p* = 0.004), comorbidity (*p* = 0.001), and distant metastasis (*p* < 0.001). Body mass index, preoperative hemoglobin, preoperative albumin, preoperative carcinoma embryonic antigen, tumor location, neoadjuvant chemoradiotherapy, smoking, history of abdominal surgery, and open surgery did not significantly change the risk of TI non-closure.

**Conclusion:**

We identified five preoperative risk factors for TI non-closure after sphincter-preserving surgery for rectal cancer. This information enables surgeons to identify high-risk groups before surgery, inform patients about the possibility of PS in advance, and consider performing protective colostomy or Hartmann surgery.

**Supplementary Information:**

The online version contains supplementary material available at 10.1186/s12957-024-03363-z.

## Introduction

Anastomotic leak (AL) is the most serious complication following rectal cancer surgery, with an incidence of 3-20% [1–3]. Among these cases, 10-35% require reoperation [4], and approximately 2% of patients die after AL [5]. Moreover, AL may increase the risk of local tumor recurrence after rectal cancer surgery [6, 7]. Protective temporary ileostomy (TI) is the simplest and most effective treatment to reduce the severity of AL [8, 9]. Fecal diversion can significantly reduce the incidence of AL, shorten hospital stay, and reduce the rate of emergency reoperation. Simultaneously, should AL occur, the associated peritonitis symptoms and systemic inflammatory response are markedly reduced [9, 10]. Therefore, many surgeons routinely incorporate TI into sphincter-preserving surgery for rectal cancer.

However, an ileostomy is associated with specific complications such as parastomal hernia, intestinal obstrcutions, periostomy dermatitis, high-output dehydration, acute renal impairment, and electrolyte balance disturbance [11, 12]. In addition, several studies [13–16] have shown that 6–23% of TI will not be reversed due to tumor recurrence, anastomosis-related complications, and poor anal function recovery, resulting in the formation of a permanent stoma (PS). This outcome is closely related to preoperative clinical decision-making. In contrast, studies [17] have shown that ileostomy should be the procedure of choice for short-term temporary stoma, but colostomy is more suitable for patients who need long-term or permanent stoma. In patients with ileostomy, electrolyte disturbance and chronic renal impairment due to high output dehydration and peristomy dermatitis are significantly higher than colostomy, which significantly affects quality of life (QoL) in patients with persistent stoma status [18–20]. Therefore, early identification of high-risk patients unable to reverse TI is crucial for preoperative consultation and surgical planning, with colostomy or Hartmann surgery offering potential benefits for long-term prognosis. While several studies [16, 21] have explored the risk factors and causes of stoma non-closure, systematic and comprehensive assessments of preoperative risk factors and cumulative incidence of TI non-closure following rectal cancer surgery are still lacking.

The objective of this study was to explore the preoperative risk factors and cumulative incidence of TI non-closure and conversion to PS after sphincter-preserving surgery for rectal cancer, utilizing a systematic review and meta-analysis.

## Methods

This systematic review and meta-analysis adhered to the guidelines outlined in the Preferred Reporting Items for Systematic Reviews and Meta-analyses (PRISMA, Supplementary Digital Content 1.) [22] and Assessing the Methodological Quality of Systematic Reviews (AMSTAR) [23]. Additionally, it was registered with the International Center for Prospective Systems Review (PROSPERO: CRD42023476511).

### Search strategy and selection criteria

Comprehensive searches were conducted on Web of Science, Embase, and MEDLINE databases for articles published in English while simultaneously viewing the references of papers. The search strategy included the following keywords: “ileostomy,” “permanent stoma,” “non-closure”, “nonreversal,” “no closure,” and “rectal cancer” (Supplementary Table 1). The search covered the period from the inception of the database to November 2023, as well as the language was limited to English. All identified literature was imported into Endnote 20 software for screening.

### Selection criteria

Inclusion criteria: (1) study design: prospective or retrospective cohort study; (2) study participants: patients with rectal cancer who underwent sphincter-preserving surgery for rectal cancer and TI; and (3) study content: exploration of preoperative risk factors for TI non-closure after rectal cancer surgery.

Exclusion criteria: (1) letters, meetings, comments, trial protocols, and articles that were not available in full text; (2) studies that did not provide raw data; and (3) studies with higher quality and more detailed data selected for studies with repeated cases.

### Study selection and data extraction

The pulled studies were imported into Endnote20 software for initial screening by reviewing the title and abstract. The remaining study underwent a second screening by reviewing the full text to determine the studies that were included in the meta-analysis. All steps were conducted by two authors, and in cases of disagreement, a third author was consulted. The following elements were extracted (1), study characteristics: author, publication year, study area, study period, and study type, number of cases, sex ratio, grouping method, and age (2). risk factors: sex, age, body mass index (BMI), comorbidities, American Society of Anesthesiologists (ASA) score, smoking, tumor location, metastasis, surgical method, history of abdominal surgery, neoadjuvant chemotherapy (NCT) and neoadjuvant radiotherapy (NRT), preoperative hemoglobin (Hb), preoperative albumin (Alb), and preoperative carcinoma embryonic antigen (CEA). In cases of a lack of information, we made efforts to contact the author by email or phone.

### Quality assessment

The Newcastle-Ottawa Scale (NOS) [24] was used to evaluate retrospective cohort studies and assess the risk of bias of each study in the following three areas: patient representation, exposure and outcome determination, and adequacy of follow up. The total NOS score ranges from 0 to 9, and studies with scores ≥ 7 are considered high-quality studies, those with scores 4–6 are considered moderate-quality studies, and those with scores ≤ 3 are considered low-quality studies. Quality evaluation was carried out by two authors, and differences were resolved through discussion.

### Statistical analysis

RevMan 5.3 statistical software provided by the Cochrane Collaboration was used to perform the meta-analysis. Data are reported as the combined weighted mean difference (WMD) for continuous variables and odds ratio (OR) for categorical variables. If the data in the original study were not represented as mean and standard deviation (SD), conversion to mean or SD was performed before meta-analysis [25, 26]. All effect sizes were expressed as 95% confidence interval (CI). I² was used to analyze the heterogeneity of the studies. A funnel plot was used to determine whether bias existed in the results. If there was publication bias or other biases, a sensitivity analysis of the results was performed to determine whether the results were stable and reliable.

## Results

### Selection of the included studies

**Fig. 1:**
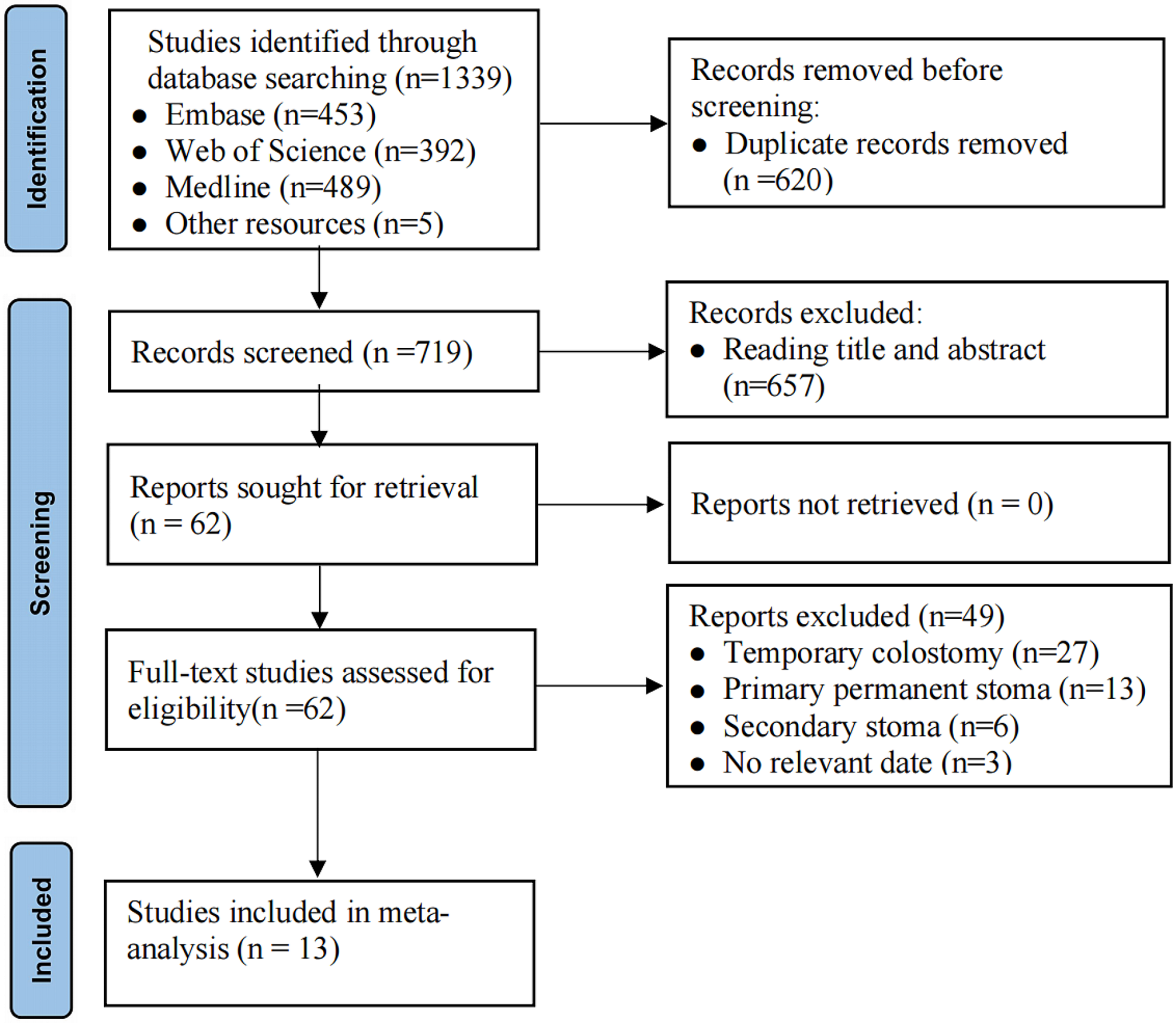
Flowchart of the search strategy

A total of 1605 studies in English were initially retrieved, and five additional studies were supplemented from other sources. After deduplication using Endnote20 software, 719 studies remained. Titles and abstracts were reviewed, and studies not meeting the inclusion criteria were excluded, leaving 62. After a full-text review, 13 studies [27–39] were included in the meta-analysis (Fig. 1). The included studies were retrospective cohort studies, encompassing 3026 patients, with 459 (15.17%) experiencing TI non-closure, subsequently converted to PS after surgery.

### Study characteristics and quality evaluation

**Fig. 2:**
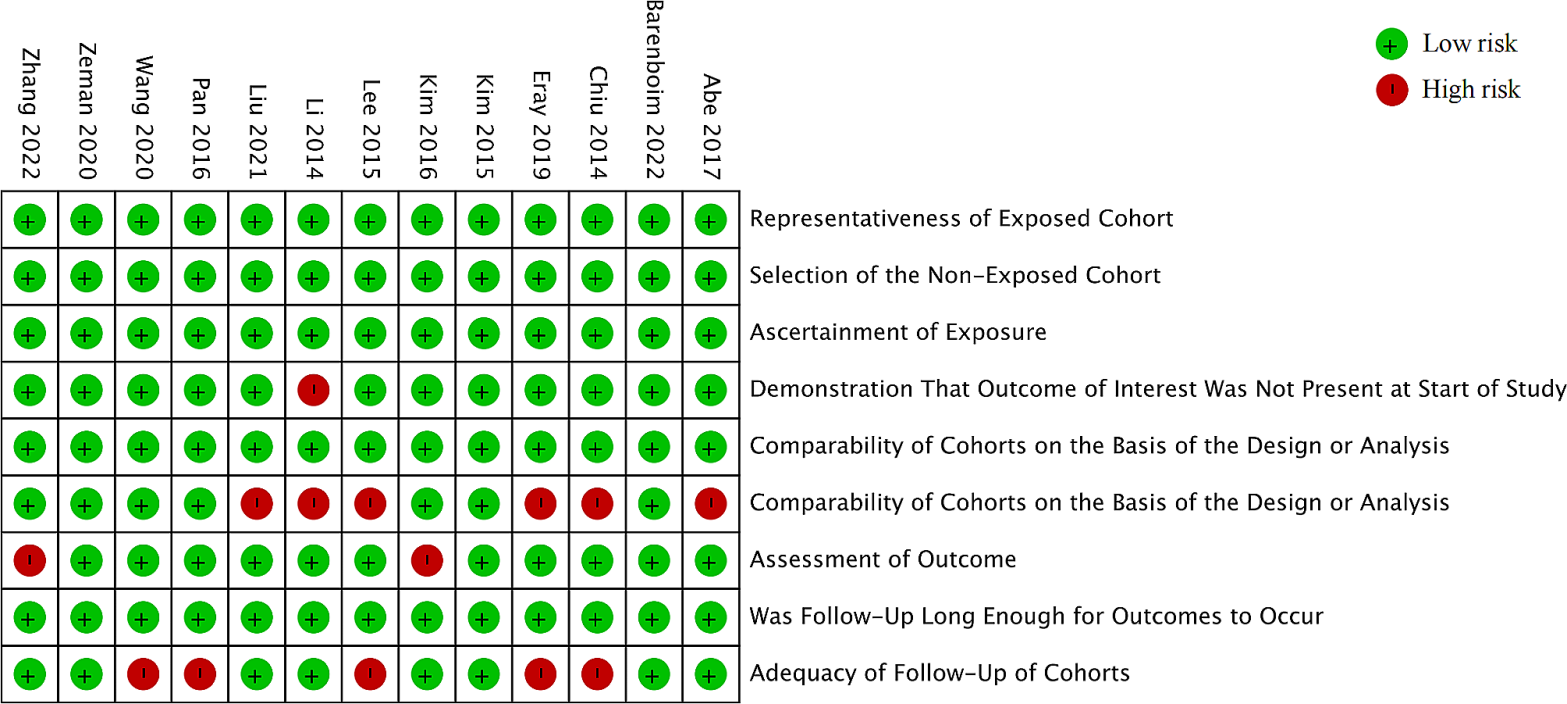
Risk or bias graph

The baseline characteristics and quality evaluations are shown in Table 1. The score for each article included in this study ranged between 7 and 9, indicating sufficient study quality (Fig. 2). The meta-analysis results for risk factors are presented in Table 2.

**Table 1:** Studies characteristics and quality

Study	Country	Study design	Period	Patients C/NC	Sex(male) C-NC	Age(years) C-NC	NOS grade
Abe 2017	Japan	Cohort study	2012–2015	25/91	84	NA	8
Barenboim 2022	Germany	Cohort study	2000–2018	25/211	12/88	65 (34–83)/62 (33–82)	9
Chiu 2014	Canada	Cohort study	2006–2012	24/138	19/100	66 ± 11/61.7 ± 11.11	7
Eray 2019	Turkey	Cohort study	2015–2018	12/54	10/40	61.67 ± 12.0/58.1 ± 10.9	7
Kim 2015	Korea	Cohort study	2004–2011	23/112	15/76	66 ± 11/61 ± 10	9
Kim 2016	Korea	Cohort study	2001–2008	64/609	49/390	58.5 ± 11.2/59.7 ± 10.0	8
Lee 2015	Korea	Cohort study	2000–2009	28/203	16/138	more than 65 year (21/139)	7
Li 2014	China	Cohort study	2014–2017	58/220	46/147	74.5 ± 5.02/73.79 ± 4.60	7
Liu 2021	China	Cohort study	2012–2019	34/232	NA	NA	8
Zhu 2022	China	Cohort study	2013–2019	52/368	38/250	62.2 ± 7.8/64.6 ± 9.6	8
Pan 2016	China	Cohort study	2006–2013	51/245	31/145	59(33–77)/59(22–87)	8
Wang 2016	China	Cohort study	2008–2018	51/230	33/152	more than 65 year (33/179)	8
Zeman 2020	Poland	Cohort study	2008–2018	38/63	30/37	61.98 ± 10.6/61.61 ± 10.81	9
Zhang 2022	China	Cohort study	2011–2019	26/159	20/92	62(32–81)/59(25–81)	8

C: Closure; NC: Non-Closure; NOS: Newcastle-Ottawa Scale; NA: Not Available

**Table 2:** Outcomes of meta analysis

Risk factors	No. of studies	No. of Patients	Heterogeneity		Statistical model	Statistical method	Effect estimate [95%CI]	*P*
			I^2^	P				
**Patient-related factors**								
Age (years)	9	2131	0%	0.69	Fixed-effects	WMD	1.21[0.10, 2.31]	0.03*
Age (≥ 65 years old)	6	1256	40%	0.14	Fixed-effects	OR	1.40[1.03, 1.91]	0.03*
Sex (male)	11	2644	13%	0.32	Fixed-effects	OR	1.37[1.08, 1.73]	0.009*
BMI (kg/m²)	6	1438	0%	0.87	Fixed-effects	WMD	-0.11[-0.67, 0.44]	0.69
Smoking	2	954	0%	0.56	Fixed-effects	OR	1.21[0.10, 2.31]	0.10
Comorbidity	4	1271	0%	0.64	Fixed-effects	OR	1.73[1.24, 2.40]	0.001*
ASA score ≥ 3	10	2543	57%	0.01	Random-effects	OR	2.13[1.27, 3.55]	0.004*
**Tumor-related factors**								
Location (≤ 5 cm)	5	1598	0%	0.55	Fixed-effects	OR	1.22 [0.90, 1.65]	0.20
Location (cm)	3	1094	0%	0.52	Fixed-effects	WMD	-0.45[-0.95, 0.67]	0.09
Metastasis	6	1157	50%	0.07	Random-effects	OR	5.94[3.10,11.39]	< 0.001*
Preoperative Hb (g/dL)	2	463	0%	0.99	Fixed-effects	WMD	-0.61 [-1.56, 0.33]	0.20
Preoperative Alb (g/dL)	2	463	0%	0.47	Fixed-effects	WMD	-1.21[-5.95, 3.54]	0.62
CEA (ng/ml)	2	251	25%	0.25	Fixed-effects	WMD	1.44[-15.17, 18.05]	0.87
**Treatment-related factors**								
NRT	9	2477	23%	0.24	Fixed-effects	OR	1.07 [0.82, 1.41]	0.21
NCT	5	996	0%	0.60	Fixed-effects	OR	0.76 [0.50, 1.16]	0.20
History of abdominal surgery	6	1732	34%	0.18	Fixed-effects	OR	1.24 [0.87, 1.76]	0.23
Open surgery	5	1018	66%	0.02	Random-effects	OR	2.26[1.09, 4.67]	0.03*
Incidence of TI non-closure	13	3026	61%	0.002	Random-effects	RD	0.16[0.13, 0.19]	< 0.001*

CI: Confidence interval; WMD: Weighted mean difference; OR: Odds ratio; BMI: Body mass index; ASA: American Society of Anesthesiologists; Hb: hemoglobin; Alb: lbumin; CEA: Carcinoma embryonic antigen; NRT: Neoadjuvant radiotherapy; NCT: Neoadjuvant chemotherapy; TI: Temporary ileostomy

### Data analysis

#### Patient-related factors

**Fig. 3:**
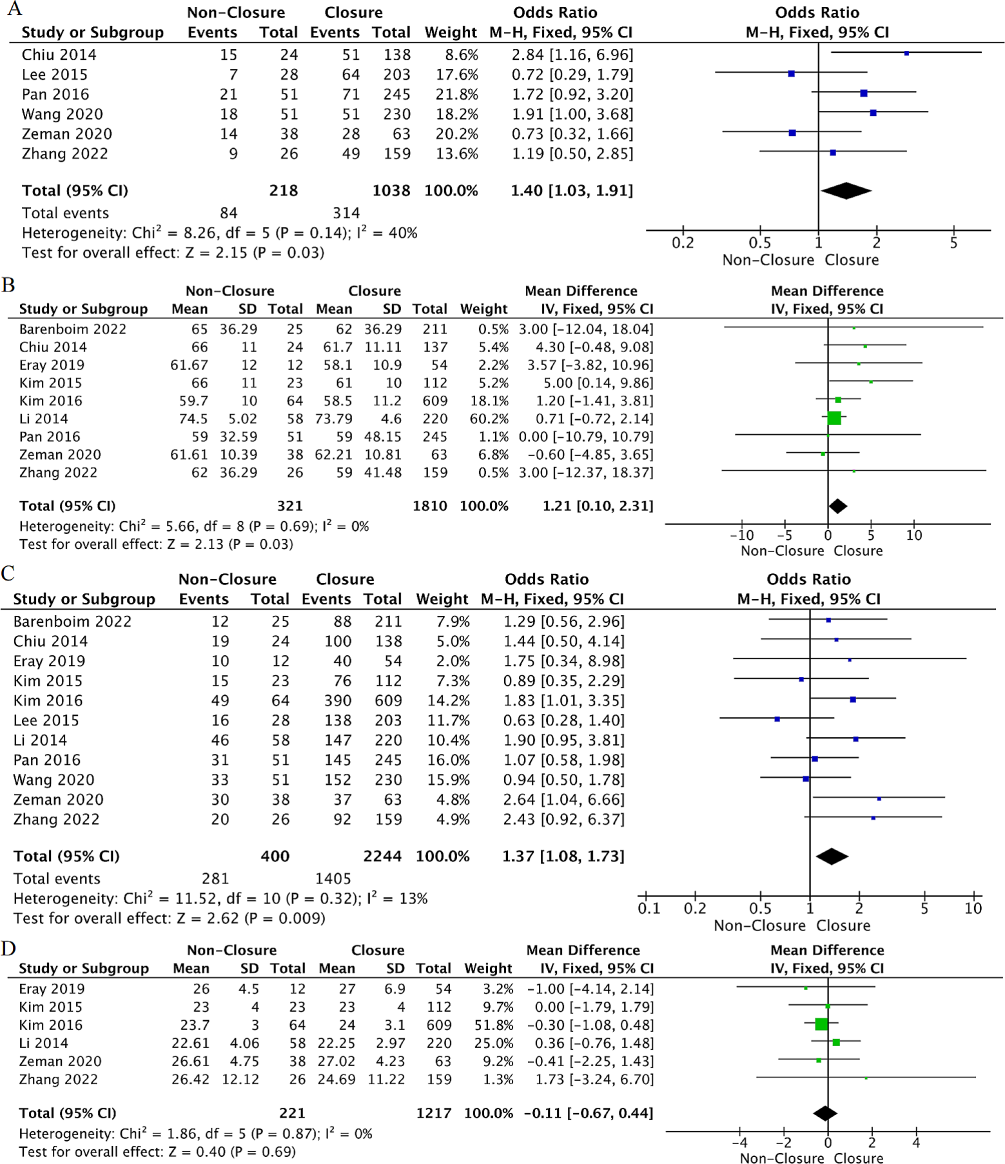
Forest plot detailing the association of patient- related factors with ileostomy non-closure after rectal cancer resection. (**A**) age > 65 years old; (**B**) age; (**C**) sex (male); (**D**) BMI

Age: Strong evidence from nine studies [28–32, 34, 36, 38, 39] explored the association between age and TI non-closure after rectal cancer surgery. The meta-analysis suggested that older patients were at greater risk for TI non-closure (WMD = 1.21, 95% CI: 0.10 to 2.31, *p* = 0.03, I²=0%). Moreover, meta-analysis results of six studies [29, 34, 36–39] revealed that patients > 65 years had a 40% increase in the risk of TI non-closure (OR = 1.40, 95%CI: 1.04 to 1.91, *p* = 0.03, I²=40%) (Fig. 3).

Sex: Strong evidence from 11 studies [28–34, 36–39] explored the association between sex and TI non-closure after rectal cancer surgery. The meta-analysis indicated that male sex was associated with a greater risk for TI non-closure (OR = 1.37, 95%CI: 1.08 to 1.73, *p* = 0.009, I²=13%) (Fig. 3).

BMI: Moderate evidence from six studies [30–32, 34, 38, 39] analyzed the association between BMI and TI non-closure after rectal cancer surgery. The pooled analysis indicated no association between BMI and the risk of TI non-closure (WMD=-0.11, 95%CI: -0.67 to 0.44, *p* = 0.69, I²=0%) (Fig. 3). In addition, there was also no association between BMI > 25 or BMI > 30 and TI non-clsoure after cancer resection from the pooled analysis (OR = 1.17, *p* = 0.41, I²=0%) (OR = 1.39, *p* = 0.38, I²=0%) (Supplementary Fig. 1).

**Fig. 4:**
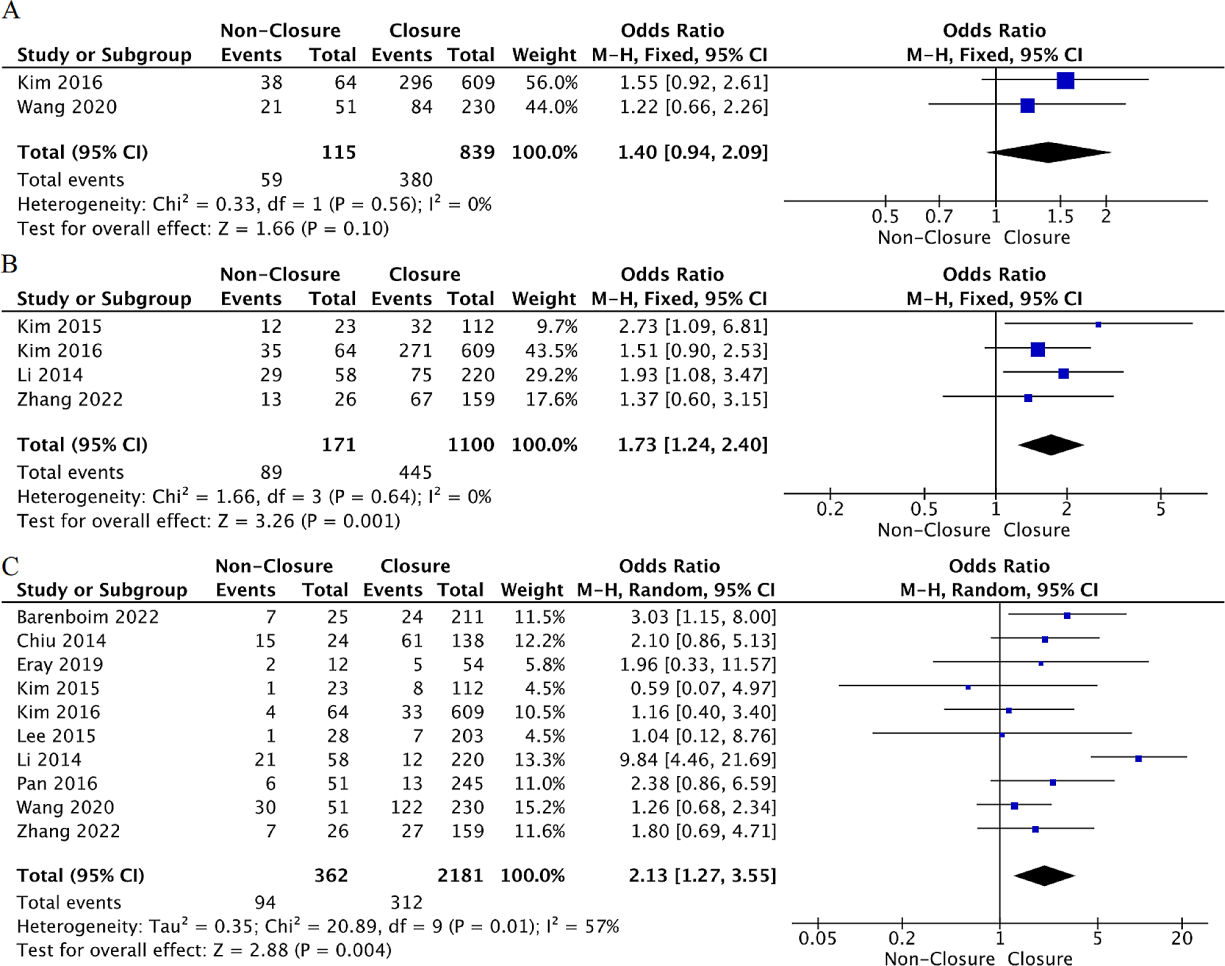
Forest plot detailing the association of patient- related factors with ileostomy non-closure after rectal cancer rescetion. (**A**) smoking; (**B**) comorbidity; (**C**) ASA score ≥ 3

Smoking: Moderate evidence from two studies [31, 37] analyzed the relationship between smoking and TI non-closure after rectal cancer resection. There was no association between smoking and the risk of TI non-closure (OR = 1.40, 95%CI: 0.94 to 2.09, *p* = 0.10, I²=0%) (Fig. 4).

Comorbidity: Moderate evidence from four studies [31, 32, 34, 39] explored the association between comorbidities and TI non-closure after rectal cancer surgery. The meta-analysis suggested that having comorbidity was associated with a 73% increase in the risk of TI non-closure (OR = 1.73, 95%CI: 1.24 to 2.40, *p* = 0.001, I²=0%) (Fig. 4).

ASA score: Strong evidence from ten studies [28–34, 36, 37, 39] explored the association between ASA scores and TI non-closure after rectal cancer surgery. The meta-analysis found that an ASA score ≥ 3 was associated with more than two-fold increased risk of TI non-closure (OR = 2.13, 95%CI: 1.27 to 3.55, *p* = 0.004, I²=57%) (Fig. 4). After sensitivity analysis, when we excluded the study of Li et al [34], the heterogeneity was markedly reduced (*p* = 0.80, I²=0%) (Supplementary Table 2). However, the results of the factor did not change; therefore, it was included in the meta-analysis, and the random-effects model was combined to verify the reliability of the results.

#### Tumor-related factors

**Fig. 5:**
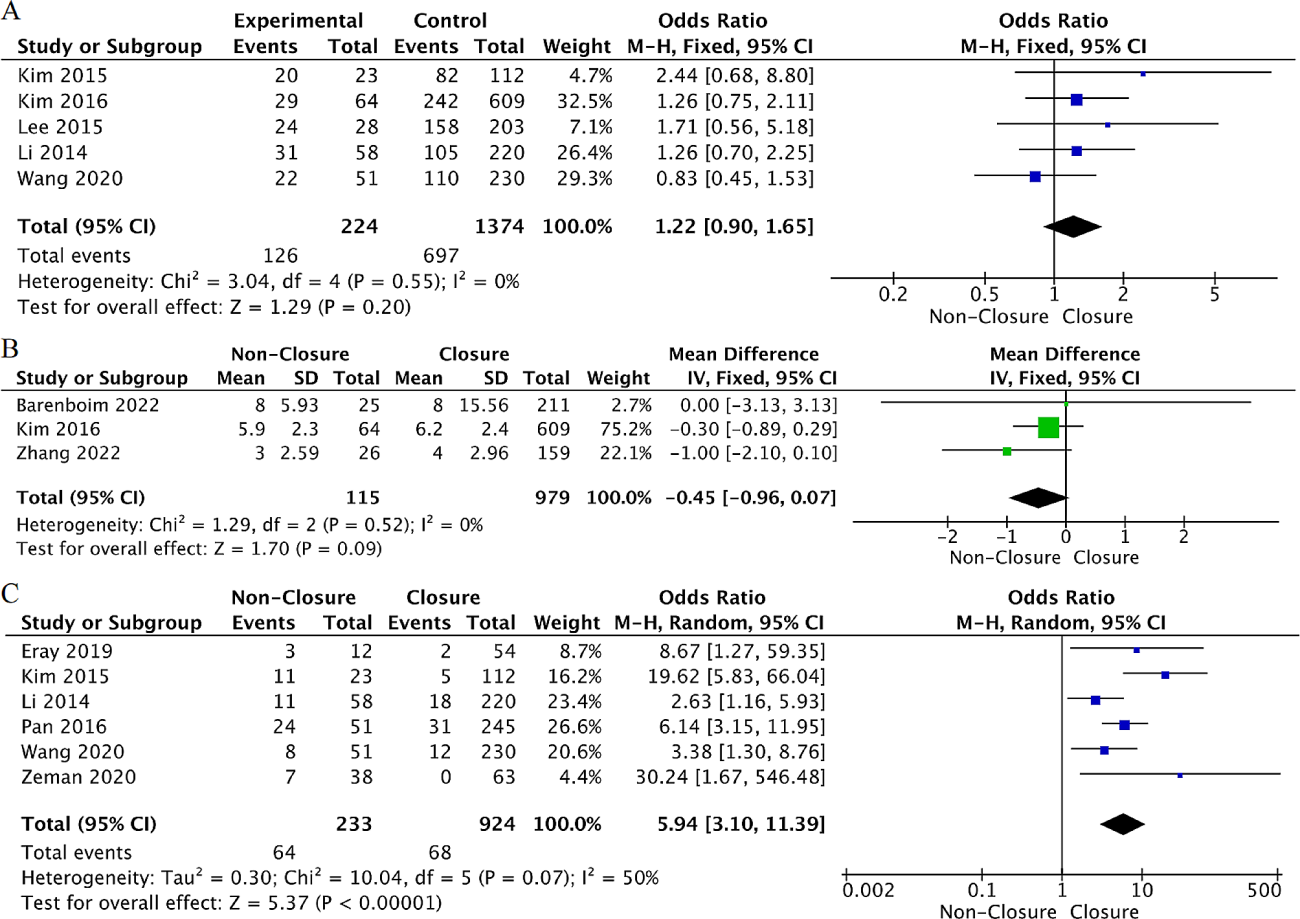
Forest plot detailing the association of tumor- related factors with ileostomy non-closure after rectal cancer rescetion. (**A**) tumor location distance < 5 cm from anus; (**B**) tumor location distance from anus; (**C**) metastasis

Location: Moderate evidence from five studies [31–34, 37] explored the relationship between tumor location and TI non-closure after rectal cancer surgery. The meta-analysis found no association between tumor location from anus < 5 cm and the risk of TI non-closure (OR = 1.22, 95%CI: 0.90 to 1.65, *p* = 0.20, I²=0%). Moreover, a pooled analysis of three studies [28, 31, 39] found that tumor location was not associated with the risk of TI non-closure. (WMD=-0.45, 95%CI: -0.90 to 0.07, *p* = 0.09, I²=40%) (Fig. 5).

Metastasis: Strong evidence from six studies [30, 32, 34, 36–38] explored the association between distant metastasis and TI non-closure after rectal cancer surgery. The pooled analysis showed that distant metastasis was associated with a nearly six-fold increased risk of TI non-closure (OR = 5.94, 95%CI: 3.10 to 11.39, *p* < 0.001, I²=50%) (Fig. 5). After sensitivity analysis, when the studies by Kim et al [32] and Li et al [34] were excluded, heterogeneity significantly reduced (*p* = 0.43, I²=0%) (Supplementary Table 2). However, the results of the factor did not change; therefore, it was included in the meta-analysis, and the random-effects model was combined to verify the reliability of the results.

Laboratory test: Moderate evidence from two studies analyzed the association between preoperative levels of Hb [34, 39], Alb [34, 39], and CEA [30, 39] and the risk of TI non-closure. No evidence was found to alter the risk of TI non-closure after rectal cancer surgery. (WMD= -0.61, *p* = 0.20; WMD= -1.21, *p* = 0.62; WMD = 1.44, *p* = 0.87) (Supplementary Fig. 2).

#### Treatment-related factors

**Fig. 6:**
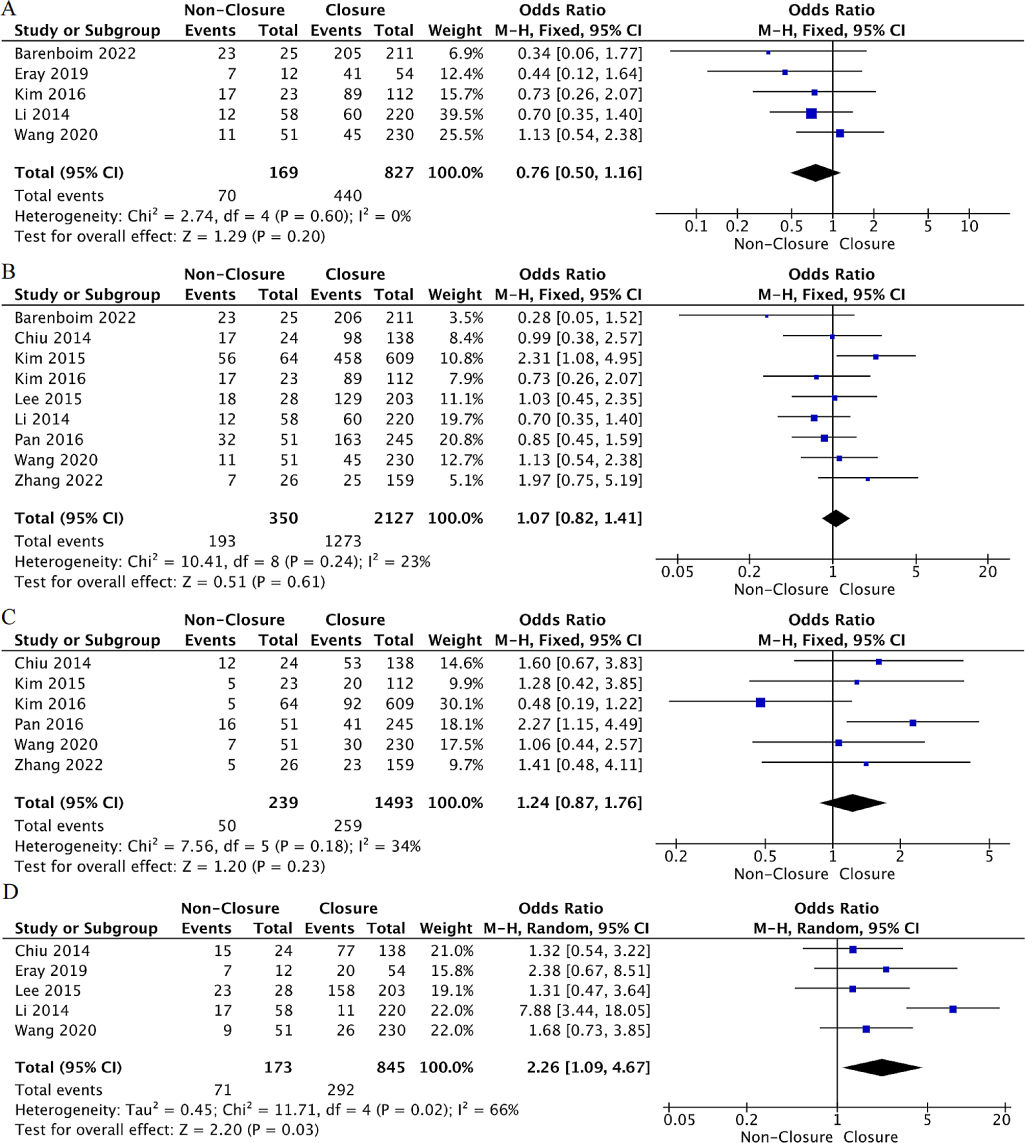
Forest plot detailing the association of treatment- related factors with ileostomy non-closure after rectal cancer rescetion. (**A**) preoperative Hb; (**B**) preoperative Alb; (**C**) preoperative CEA

NCRT: Strong evidence from five [28, 30, 31, 34, 37] and nine [28, 29, 31–34, 36, 37, 39] studies explored the relationship between NCT and NRT and the risk of TI non-closure, respectively. The meta-analysis did not find evidence that NCT or NRT increased the risk of TI non-closure after surgery (OR = 0.76, 95%CI: 0.50 to 1.16, *p* = 0.20, I²=0%; OR = 1.07, 95%CI: 0.82 to 1.41, *p* = 0.23, I²=34%) (Fig. 6).

History of abdominal surgery: Strong evidence from six studies [29, 31, 32, 36, 37, 39] explored the association between a history of abdominal surgery and TI non-closure after rectal cancer surgery. The pooled analysis indicated no association between a history of abdominal surgery and the risk of TI non-closure (OR = 1.24, 95%CI: 0.87 to 1.76, *p* = 0.23, I²=34%) (Fig. 6).

Open surgery: Moderate evidence from five studies [29, 30, 33, 34, 37] explored the association between open surgery and TI non-closure after rectal cancer surgery. The meta-analysis found that open surgery was associated with a greater risk for TI non-closure (OR = 2.26, 95%CI: 1.09 to 4.67, *p* = 0.03, I²=67%). However, after excluding the study by Li et al [34] in sensitivity analysis, the heterogeneity was significantly reduced (*p* = 0.87, I²=0%) (Supplementary Table 2), and the results changed. This change may be attributed to the fact that their study included more patients who underwent emergency surgery for obstruction or perforation, which tended to be open surgeries and resulted in serious infection-related complications. The probability of PS also tended to increase. Therefore, the study by Li et al [34] was excluded from this meta-analysis, and the final result showed that open surgery did not significantly increase the risk of TI non-closure after rectal cancer surgery (OR = 1.56, 95%CI; 0.96 to 2.52, *p* = 0.07, I²=0%) (Fig. 6).

#### Incidence of ileostomy non-closure

**Fig. 7:**
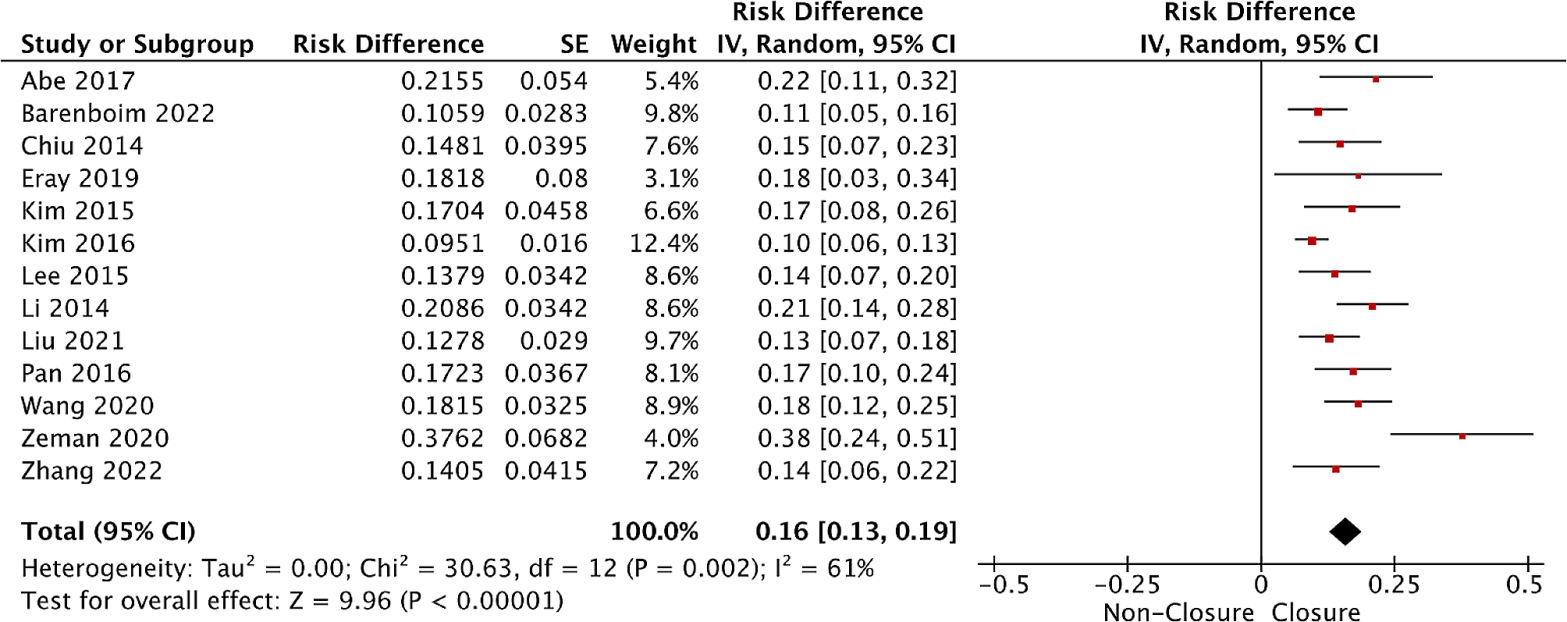
Meta-analysis of pooled data on the incidence of AS

Strong evidence from thirteen studies [27–39] has reported the occurrence of TI non-closure after rectal cancer surgery. The results of the meta-analysis showed that the incidence of TI non-closure was 16% (95%CI: 13–19%, I²=61%) (Fig. 7).

### Publication bias

A funnel plot of male sex was used to identify any evidence of publication bias. The two sides of the funnel plot were approximately symmetrical, suggesting that there was no evidence of publication bias in this study (Fig. 8). The funnel plots of the other factors are presented in Supplementary Figs. 3–13.

**Fig. 8:**
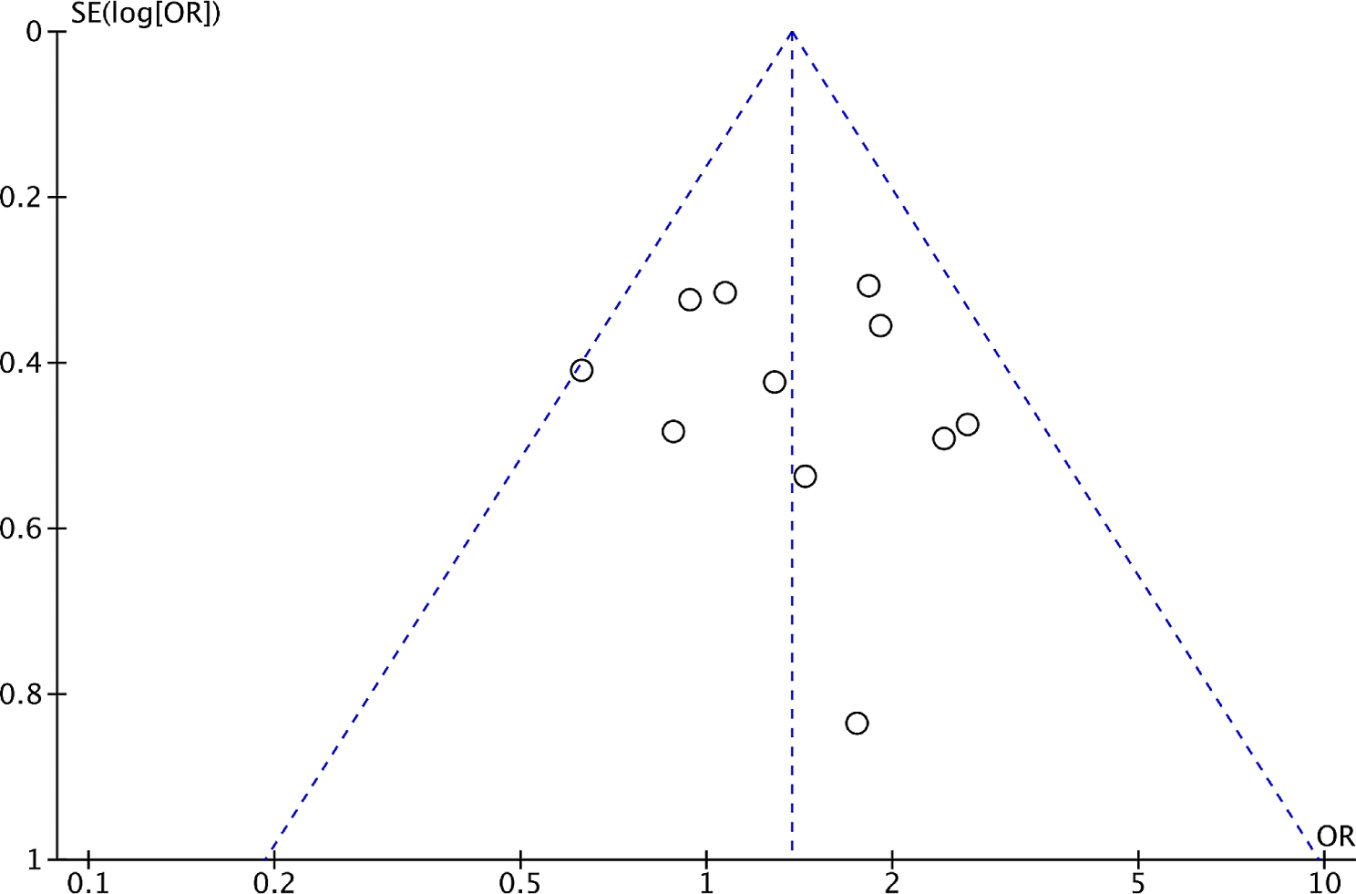
Funnel plot of the male sex

### Sensitivity analysis

The meta-analysis indicated obvious heterogeneity in several risk factors, including ASA score (I²=57%, *p* = 0.01), distant metastasis (I²=50%, *p* = 0.07), and open surgery (I²=66%, *p* = 0.02). Sensitivity analysis excluded the study by Li et al. on open surgery, which markedly reduced the heterogeneity among studies and changed the results of the meta-analysis. Therefore, we excluded the studies by Li et al. from the final results for open surgery. Sensitivity analysis results for other factors did not show obvious changes in heterogeneity or the results of meta-analysis; therefore, the corresponding studies were not excluded.

## Discussion

Currently, protective ileostomy is the most widely used procedure for reducing AL after rectal cancer surgery. The decision to perform protective ileostomy is influenced by factors such as tumor location, neoadjuvant therapy, and the general condition of the patient [40]. However, approximately 6–23% of patients with TI never experience closure, significantly impacting their quality of life. Early identification of high-risk groups for TI non-closure is important to guide preoperative decision-making. We conducted a meta-analysis of 13 studies with available data to identify risk factors for TI non-closure and conversion to PS after rectal cancer surgery. Five risk factors for TI non-closure were identified, namely, older age (> 65 years old), male sex, ASA score ≥ 3, comorbidity, and distant metastasis. BMI, preoperative Hb level, preoperative Alb level, preoperative CEA level, tumor location, NCRT, smoking, history of abdominal surgery, and open surgery did not significantly alter the risk. In addition, the incidence of TI non-closure after rectal cancer surgery was 16% (95%CI: 13–19%).

### Patient-related factors

These results indicate that the risk of TI non-closure increases with age. In particular, patients aged > 65 years had a 40% increased risk of TI non-closure, which is consistent with past research [14, 16]. On one hand, this may be due to elderly patients having more underlying diseases and being weakened after primary surgery, leading to reluctance to undergo ileostomy closure surgery again. On the other hand, elderly patients are more prone to developing AL, anastomotic stenosis (AS), fecal incontinence, pelvic septicemia, and other complications after rectal cancer surgery [41, 42]. It is foreseeable that these complications significantly increase the risk of TI non-closure. In addition, elderly patients have lower QoL requirements, and some are accustomed to the lifestyle of a stoma and unwilling to pay for ileostomy closure surgery. In terms of sex, we found that male patients were at a greater risk for TI non-closure. Several studies [43, 44] have shown that male patients are at a higher risk of developing rectal AL and AS after rectal cancer surgery. Since AL is a primary risk factor for TI non-closure, this may explain the higher risk observed in male patients.

In addition, we found that patients with comorbidities and ASA scores ≥ 3 showed a significantly increased risk of TI non-closure. Comorbidities influenced anesthesia risk, post-operative complications, and post-operative weakness in patients undergoing surgery [45]. Severe post-operative complications can lead to malnutrition, hypoproteinemia, anemia, and other diseases, reducing the possibility of a second surgery. Moreover, serious complications such as pelvic septicemia, chronic infection of the pelvic cavity, and prolonged wounds can lead to cachexia in patients. These complications have long-term and far-reaching impact on patients and increase their fear of reoperation [46, 47], which is the main reason for patients avoiding ileostomy closure surgery.

### Tumor-related factors

Our results showed that the distance of the tumor from the anus was not a risk factor for TI non-closure after surgery. However, most studies included in this meta-analysis distinguished between low rectal cancer (< 5 cm) and middle and high rectal cancers (> 5 cm). Whether sphincter-preserving surgery for ultra-low rectal cancer (< 3 cm) increases the risk of TI non-closure requires further exploration. However, in patients with preoperative distant metastasis, the risk of TI non-closure was nearly six times greater than that in patients without metastasis, which is similar to the findings of most past studies [48–50]. Clinically, patients with distant metastatic rectal cancer have a worse prognosis, higher risk of post-operative recurrence, and shorter life expectancy, and some patients are more likely to develop mechanical ileus due to secondary abdominal malignancies, all of which are risks affecting TI closure. In addition, patients with distant metastasis tend to have longer chemotherapy cycles; TI closure surgery delays chemotherapy and may lead to ileostomy-related renal impairment and water and electrolyte balance disturbances, reducing chemotherapy tolerance. Therefore, we strongly recommend protective colostomy or Hartmann’s surgery for patients with distant metastasis.

Other reported risk factors for TI non-closure include preoperative nutritional status, preoperative fibrinogen concentration, and socioeconomic status. However, owing to the lack of relevant studies and data, this meta-analysis could not be further analyzed. Zeman et al. [38] suggested that a high plasma fibrinogen concentration before surgery may be an independent risk factor for TI non-closure. They found that plasma fibrinogen accelerated tumor progression and increased the risk of post-operative infection, AL, and other inflammatory reactions, which were the reasons for its influence on TI closure. Zafar et al. [51] showed that stoma closure was correlated with race, insurance type, and income status. They found that white patients had higher rates of closure than black patients, privately insured patients had higher rates of reversal than uninsured patients, and household income among those in the top quartile had higher rates of reversal than those in the bottom quartile. Future studies should provide a more comprehensive preoperative assessment of these risk factors.

### Incidence of TI non-closure

The meta-analysis results revealed that the incidence of TI non-closure after sphincter-preserving surgery for rectal cancer is approximately 16%; that is, approximately 1 out of 6 people experience TI non-closure and convert to PS. In most studies, the majority of TIs are performed within 6 months of the operation, and if TI closure is not achieved at least 1 year, it is defined as TI non-closure. However, some reseachers, such us Kim et al. [30] and Lee et al. [31], used follow-up time as a node which extended the time to define the TI non-closure to determine whether patients had a PS. This may be the main reason for the difference in the rate of TI non-closure in the existing studies.

Therefore, preoperative imaging staging should be strengthened in patients with rectal cancer to determine the presence of distant metastases, and a careful anesthesia risk assessment should be carried out. For high-risk groups with TI non-closure, early identification of high-risk factors can lead to better treatment decisions, making it more beneficial for patients to undergo protective colostomy or Hartman surgery after sphincter-preserving surgery. This approach is more suitable for PS than ileostomy. However, it is important to consider that this choice preserves the patient’s expectations of restoring a stoma. In summary, patients benefit most from assessing the risk of PS before surgery and developing a personalized surgical strategy for each patient.

### Limitations

This study had few limitations. First, all 13 studies included in the meta-analysis were retrospective; however, they were of medium to high quality based on the quality evaluation. Second, the studies included a wide range of populations, ethnicities, and study methods, reflecting high heterogeneity, especially in the definition of TI non-closure. Nevertheless, the observed heterogenicity may be attributed to the evaluated population or study design rather than actual differences. For risk factors with high heterogeneity, we used a random-effects model to verify the reliability of the results. In addition, owing to the strict inclusion criteria, fewer articles were included in the meta-analysis, and some risk factors could not be pooled due to differences in reporting forms. This may have impacted the comprehensiveness of the study results in assessing the risk factors for TI non-closure. However, the existing meta-analysis results still hold guiding significance for developing a personalized surgical strategy for each patient with rectal cancer.

## Conclusion

We conducted a meta-analysis of 13 studies worldwide, revealing that older age, male sex, ASA score ≥ 3, comorbidity, and distant metastasis were preoperative risk factors for TI non-closure after rectal cancer surgery. The current incidence of TI non-closure and conversion to PS was 16% (95%CI, 13–19%). These findings enable surgeons to better identify high-risk individuals before surgery, inform patients about the possibility of PS, and develop personalized surgical strategies to minimize the incidence of permanent ileostomy by selecting protective colostomy or Hartmann surgery. In the future, large and rigorously designed randomized controlled trials are warranted to further explore more comprehensive preoperative risk factors, including ultra-low rectal cancer and surgical methods, as well as further verify the reliability of the results of this study.

### Electronic supplementary material

Below is the link to the electronic supplementary material.

Supplementary Material 1

Supplementary Material 2

## Data Availability

No datasets were generated or analysed during the current study. All data generated or analyzed during this study are included in this published article. No additional unpublished data are available.

## Declarations

### Research registration unique identifying number

The name of the registry: RROSPERO; Research Registration Unique Identifying Number. (UIN): CRD42023476511.
