# Supplementary material for: Preoperative risk factors and cumulative incidence of temporary ileostomy non-closure after sphincter-preserving surgery for rectal cancer: a meta-analysis
Source: World J Surg Oncol. 2024 Apr 12;22:94. doi: 10.1186/s12957-024-03363-z (PMC11010286; doi:10.1186/s12957-024-03363-z)

| **Supplementary Table1. Database and search strategy of this study.** | |
| --- | --- |
| **Data bases** | **Search strategy** |
| **MEDLINE** | 1. AB=(Nonclosure OR Nonreversal OR No closure) AND AB=(Ileostomy) |
|  | 1. AB=(Permanent stoma) |
|  | 1. AB=(Rectal cancer) |
|  | 4. (1 OR 2) AND 3 |
| **Embase** | #1 ‘Ileostomy’:ab,ti |
|  | #2 ‘Nonclosure’:ab,ti OR ‘Nonreversal’:ab,ti OR ‘No closure’:ab,ti |
|  | #3 #1 AND #2 |
|  | #4 ‘Permanent stoma’:ab,ti |
|  | #5 ‘Rectal cancer’:ab,ti |
|  | #6 (#3 OR #4) AND #5 |
| **Web of Science** | 1. AB=(Nonclosure OR Nonreversal OR No closure) AND AB=(Ileostomy) |
|  | 1. AB=(Permanent stoma) |
|  | 1. AB=(Rectal cancer) |
|  | 4. (1 OR 2) AND 3 |

| **Supplementary Table 2. Outcomes of sensitivity analysis** | | | | |
| --- | --- | --- | --- | --- |
| **Sensitivity analysis** | **Heterogeneity test** | | **Overall effect** |  |
|  | **I ² (%)** | **Tau²** | **OR/WMD and 95%CI** | **p** |
| **ASA score ≥3** |  |  |  |  |
| Include all studies | 57 | 0.35 | 2.13[1.27, 3.55] | 0.004 |
| Exclude Barenboim 2022 | 61 | 0.42 | 2.01[1.13, 3.58] | 0.02 |
| Exclude Chiu 2014 | 62 | 0.45 | 2.10[1.17, 3.79] | 0.01 |
| Exclude Eray 2019 | 62 | 0.40 | 2.13[1.23, 3.68] | 0.007 |
| Exclude Kim 2015 | 59 | 0.35 | 2.26[1.34, 3.81] | 0.002 |
| Exclude Kim 2016 | 59 | 0.38 | 2.27[1.31, 3.96] | 0.004 |
| Exclude Lee 2015 | 61 | 0.38 | 2.19[1.28, 3.75] | 0.004 |
| Exclude Li 2014 | 0 | 0.00 | 1.67[1.19, 2.35] | 0.003 |
| Exclude Pan 2016 | 62 | 0.43 | 2.08[1.16, 3.70] | 0.01 |
| Exclude Wang 2020 | 52 | 0.35 | 2.34[1.34, 4.07] | 0.003 |
| Exclude Zhang 2022 | 61 | 0.43 | 2.15[1.20, 3.84] | 0.01 |
| **Metastasis** |  |  |  |  |
| Include all studies | 50 | 0.30 | 5.94 [3.10, 11.39] | <0.001 |
| Exclude Eray 2019 | 59 | 0.37 | 5.82 [2.81, 12.05] | <0.001 |
| Exclude Kim 2015 | 20 | 0.07 | 4.50[2.66, 7.61] | <0.001 |
| Exclude Li 2014 | 36 | 0.20 | 7.41[3.77, 14.54] | <0.001 |
| Exclude Pan 2016 | 59 | 0.58 | 6.34[2.54, 15.83] | <0.001 |
| Exclude Wang 2020 | 55 | 0.41 | 7.09[2.54, 15.89] | <0.001 |
| Exclude Zeman 2020 | 53 | 0.28 | 5.49[2.85, 10.57] | <0.001 |
| **Open surgery** |  |  |  |  |
| Include all studies | 66 | 0.45 | 2.26[1.09, 4.67] | 0.03 |
| Exclude Chiu 2014 | 69 | 0.53 | 2.60[1.09, 6.19] | 0.03 |
| Exclude Eray 2019 | 74 | 0.59 | 2.23[0.93, 5.36] | 0.07 |
| Exclude Lee 2015 | 71 | 0.54 | 2.57[1.09, 6.07] | 0.03 |
| Exclude Li 2014 | 0 | 0.00 | 1.56[0.96, 2.52] | 0.07 |
| Exclude Wang 2020 | 73 | 0.66 | 2.44[0.95, 6.25] | 0.06 |

**Supplementary Figure Legends**

Figure 1. Forest plot detailing the association of patient- related factors with ileostomy non-closure after rectal cancer rescetion. (A) BMI>25; (B) BMI>30.

Figure 2. Forest plot detailing the association of tumor- related factors with ileostomy non-closure after rectal cancer rescetion. (A) preoperative Hb; (B) preoperative Alb; (C) preoperative CEA.

Figure 3. Funnel plot of the age.

Figure 4. Funnel plot of the BMI.

Figure 5. Funnel plot of the ASA score.

Figure 6. Funnel plot of the comorbidity.

Figure 7. Funnel plot of the location.

Figure 8. Funnel plot of the metastasis.

Figure 9. Funnel plot of the neoadjuvant radiotherapy.

Figure 10. Funnel plot of the neoadjuvant chemotherap.

Figure 11. Funnel plot of the history of abdominal surgery.

Figure 12. Funnel plot of the open surgery.

Figure 13. Funnel plot of the incidence of TI non-closure.

Figure 1.


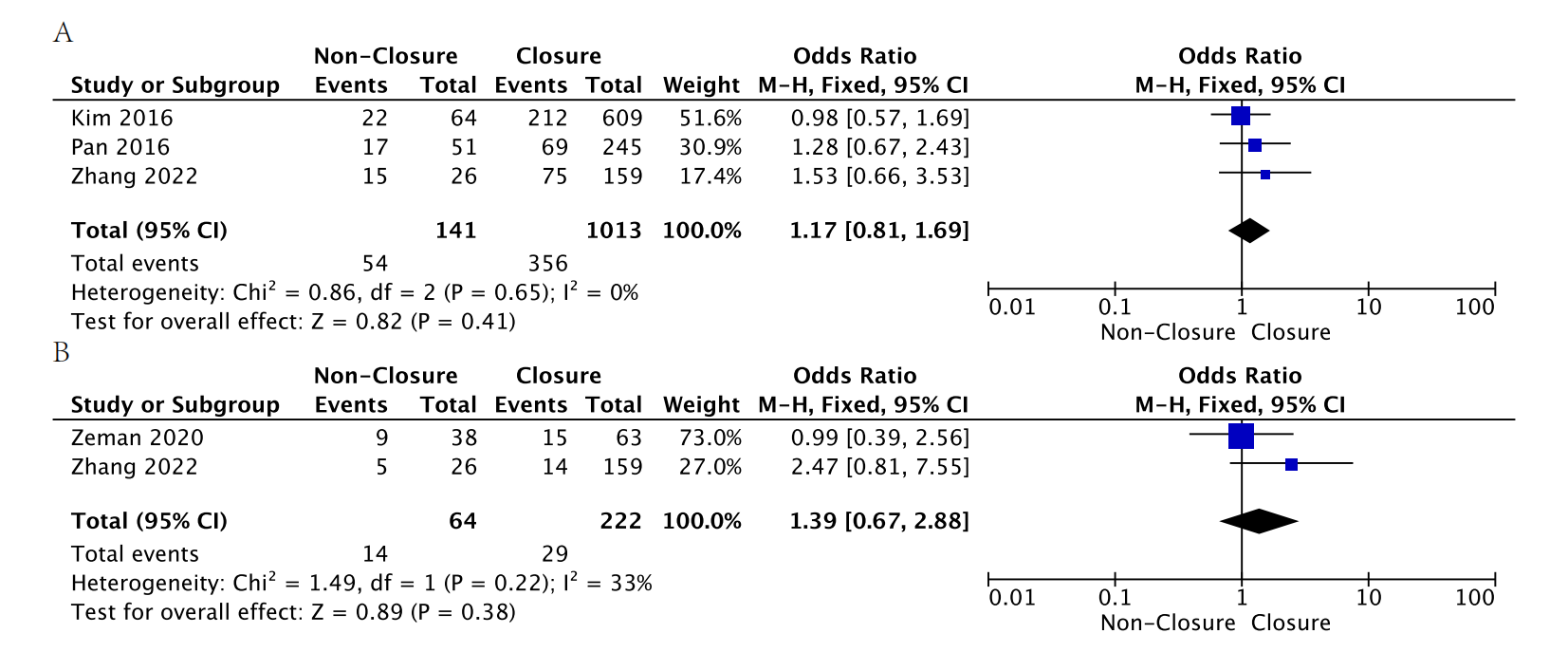


Figure 2.


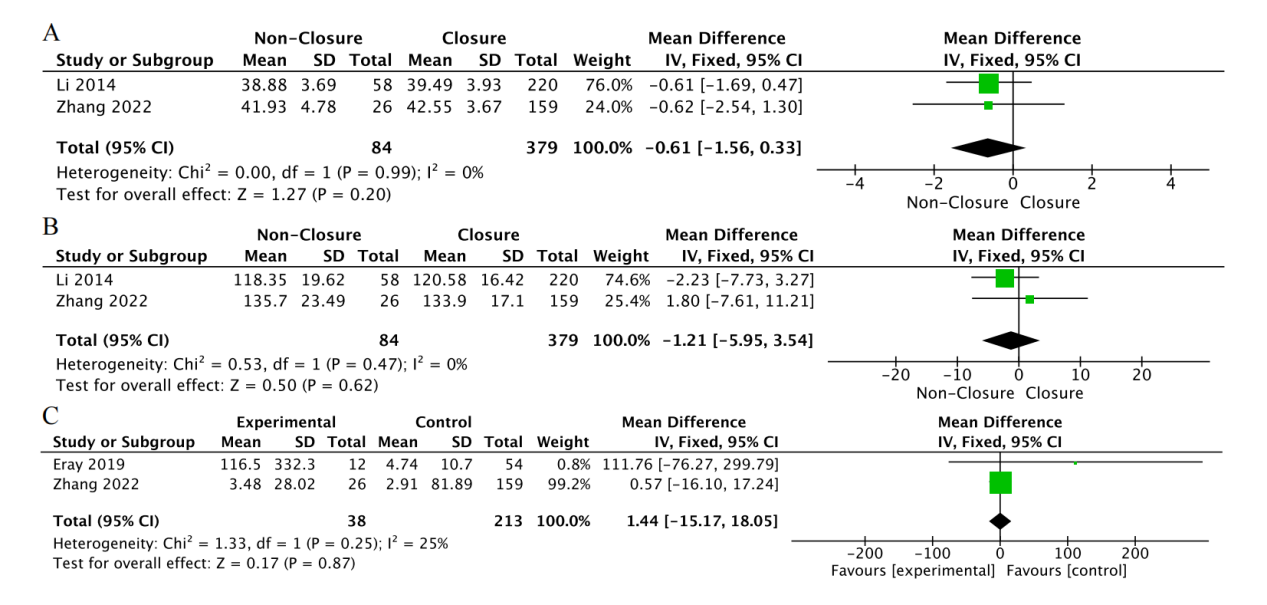


Figure 3.


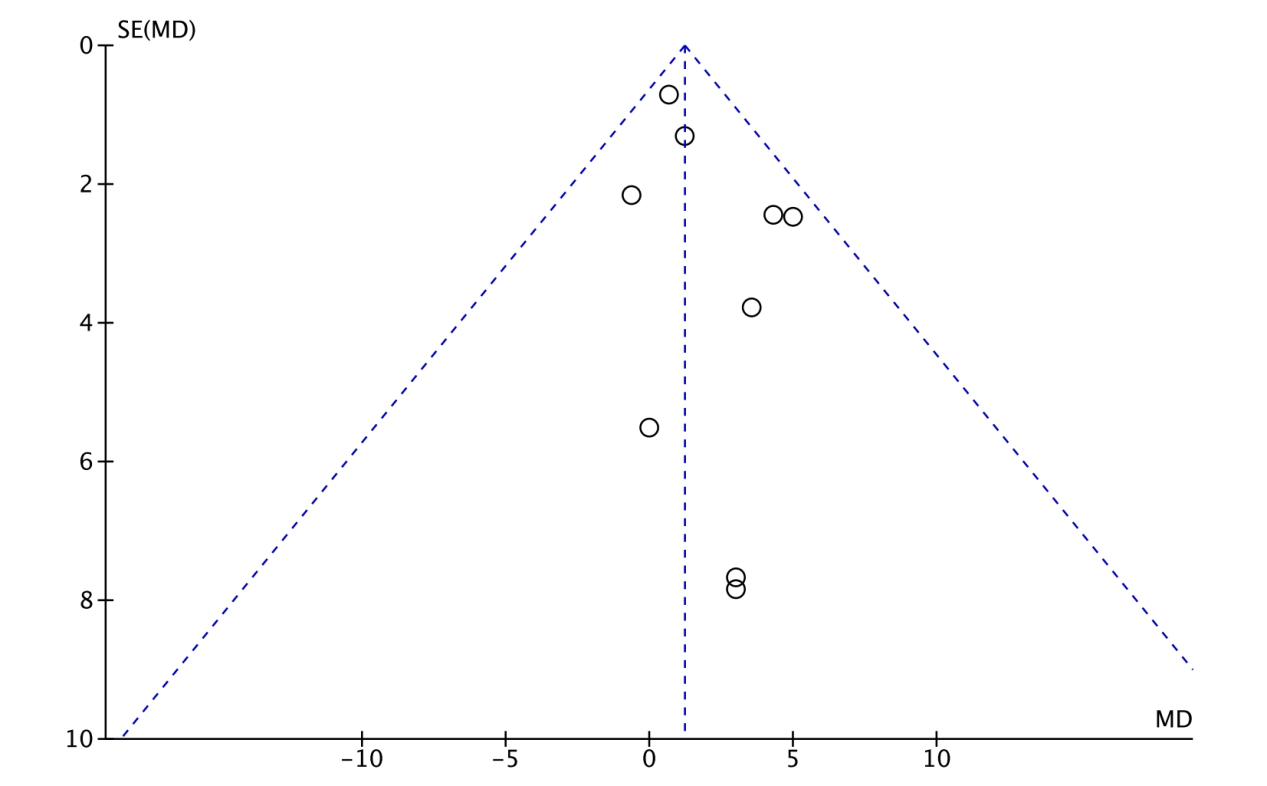


Figure 4.


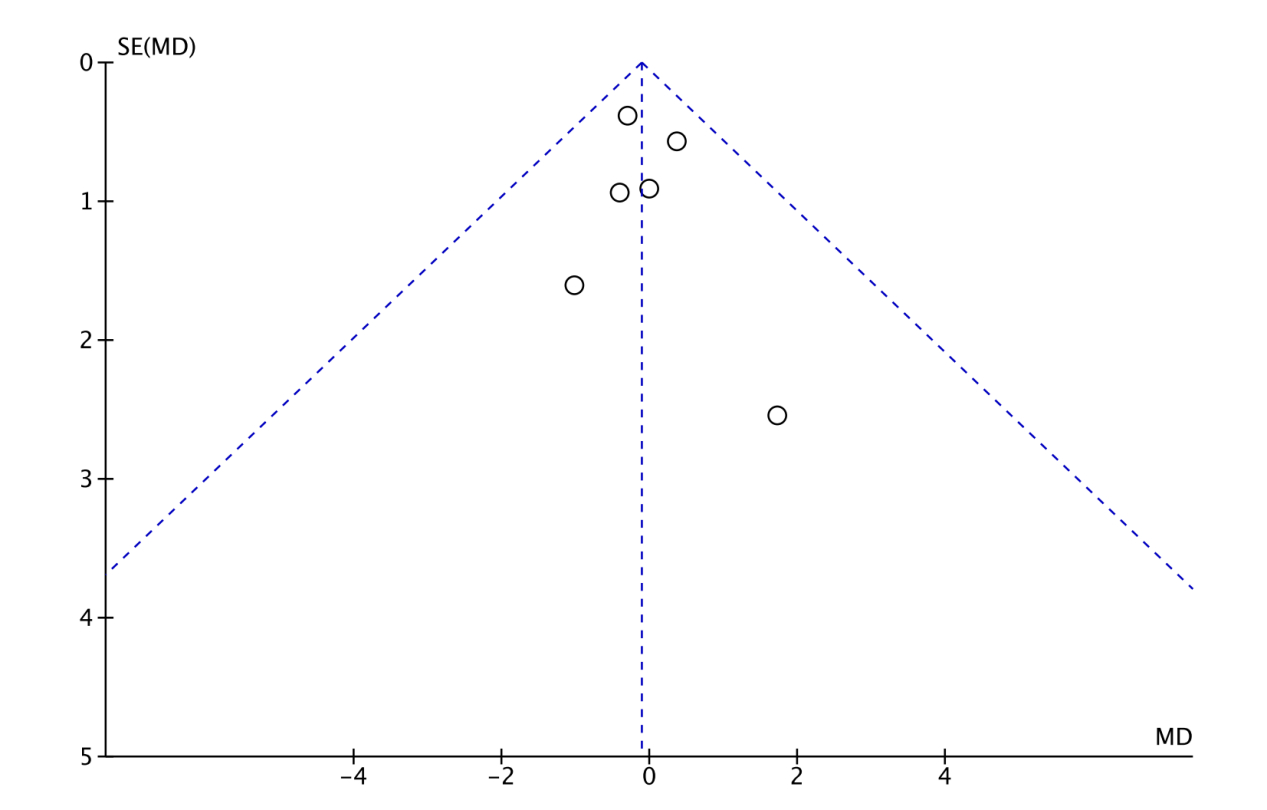


Figure 5.


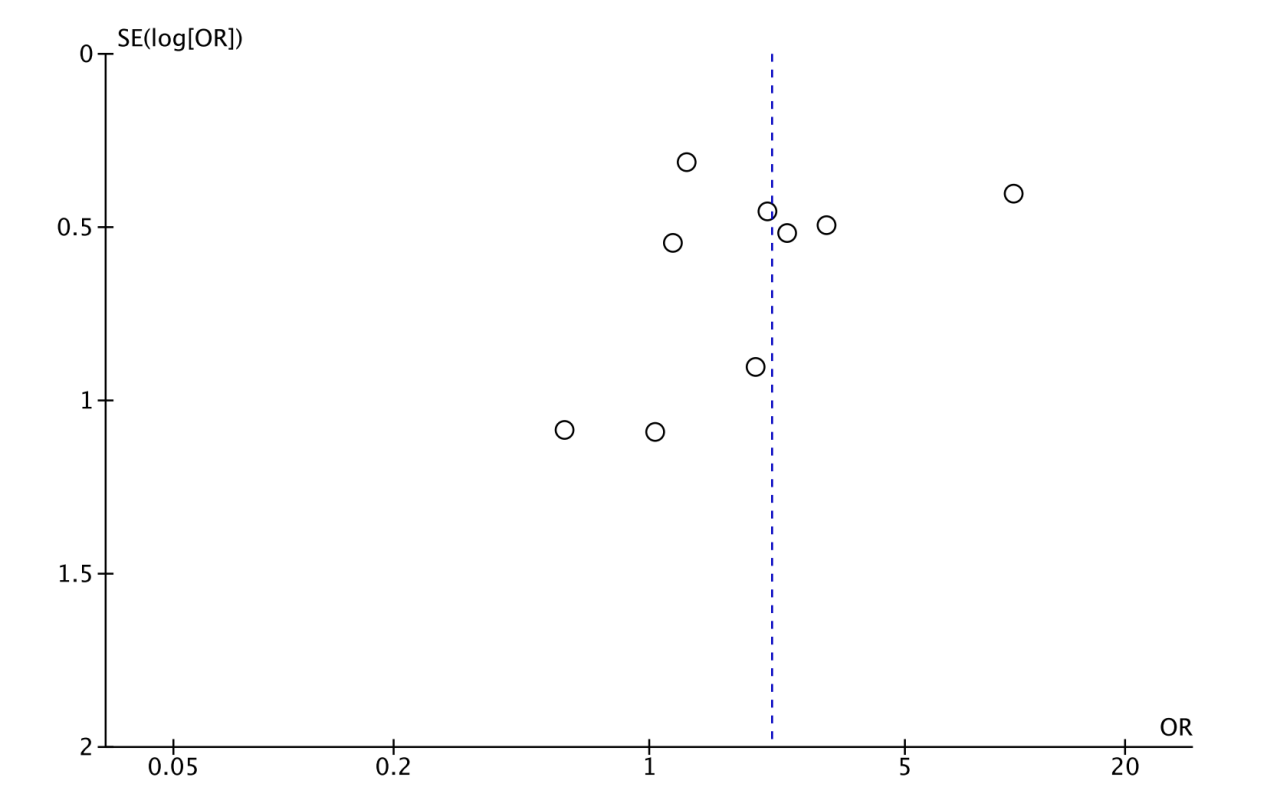


Figure 6.


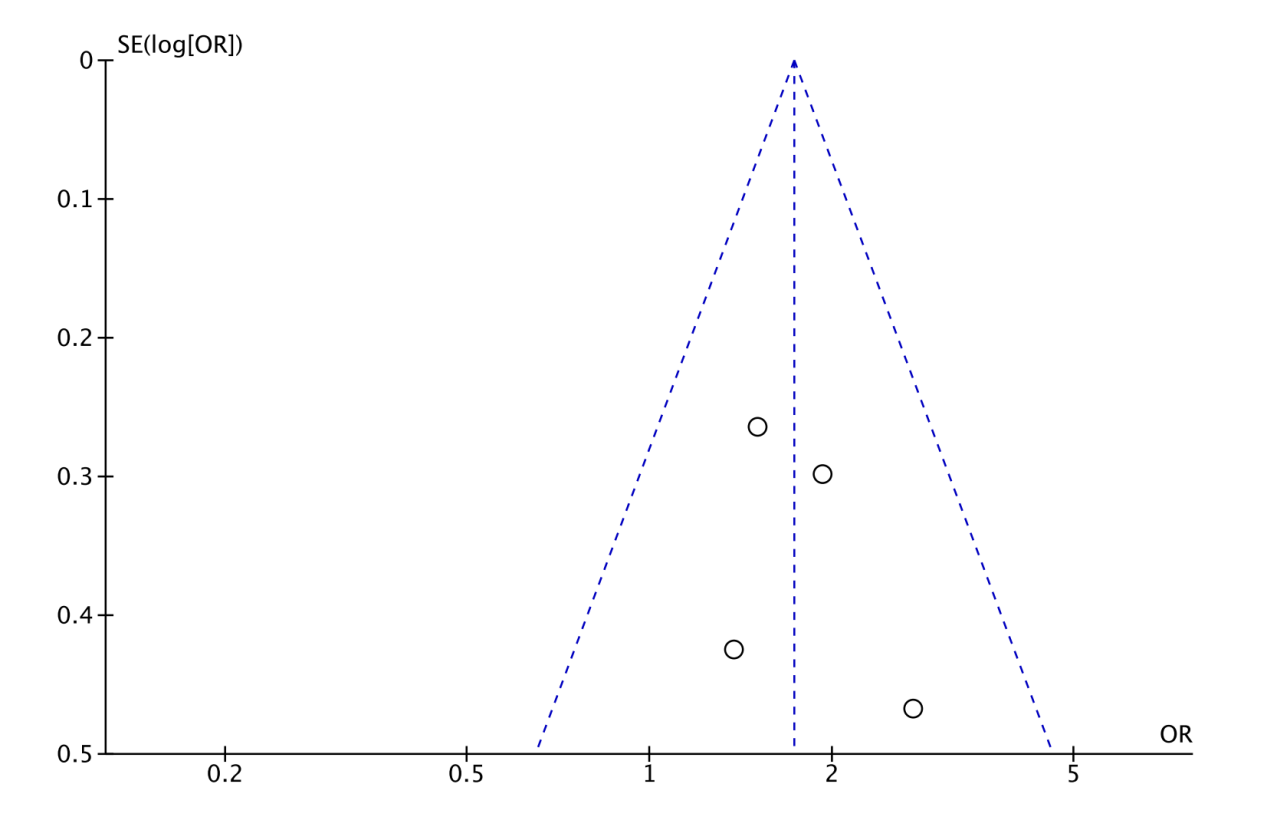


Figure 7.


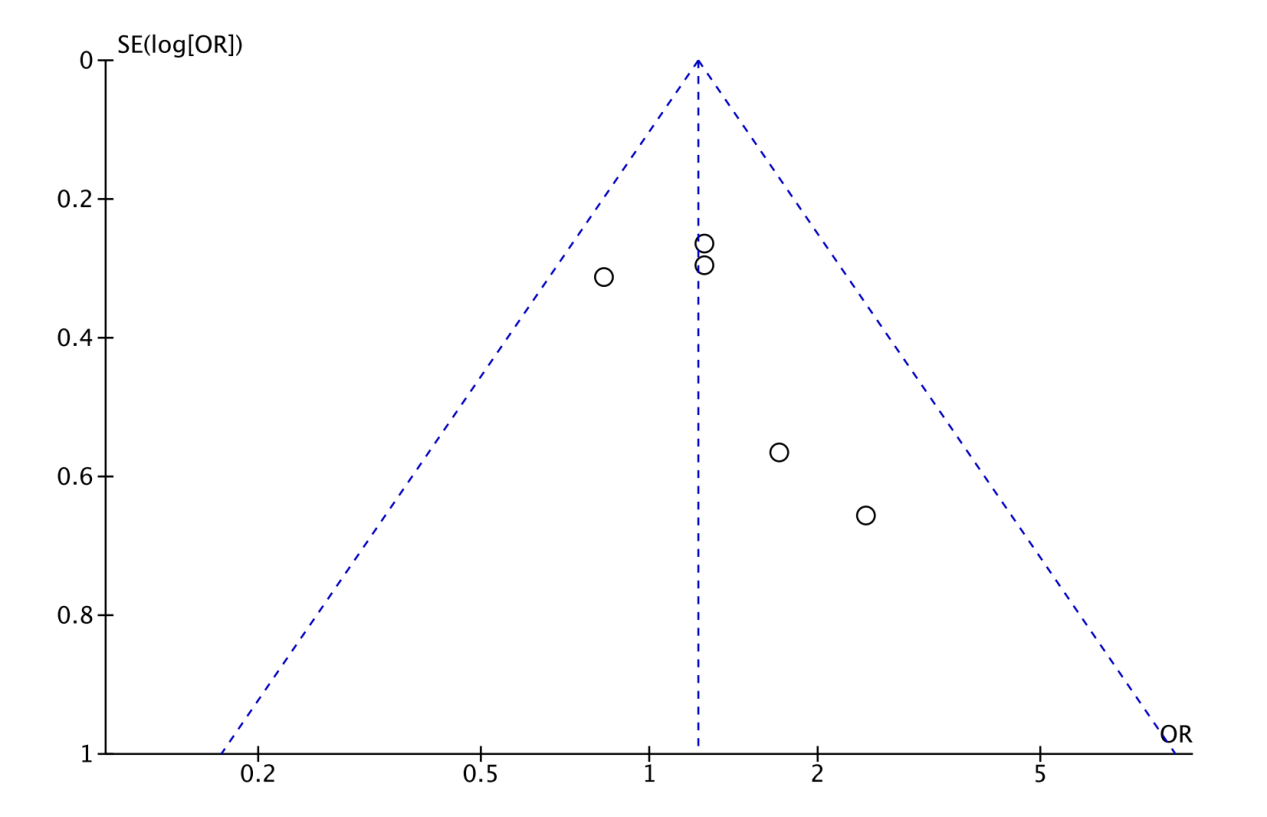


Figure 8.


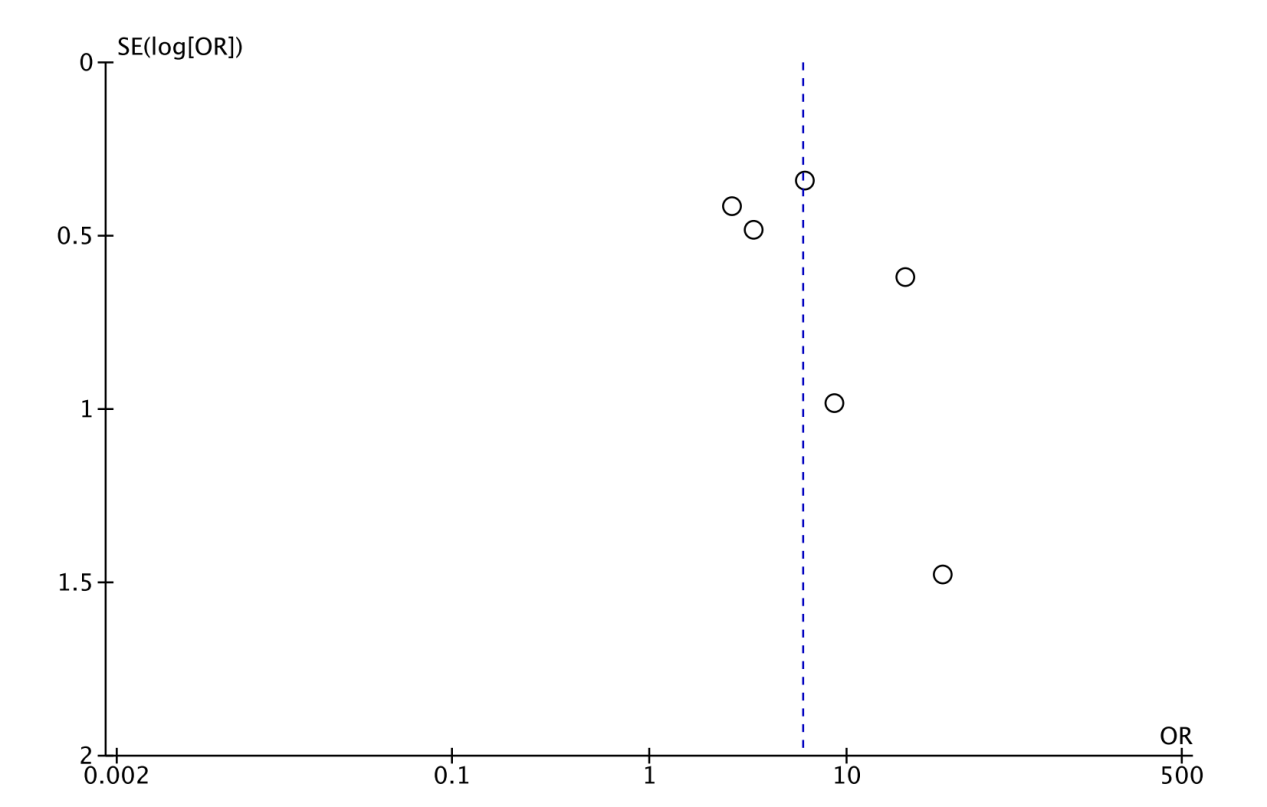


Figure 9.


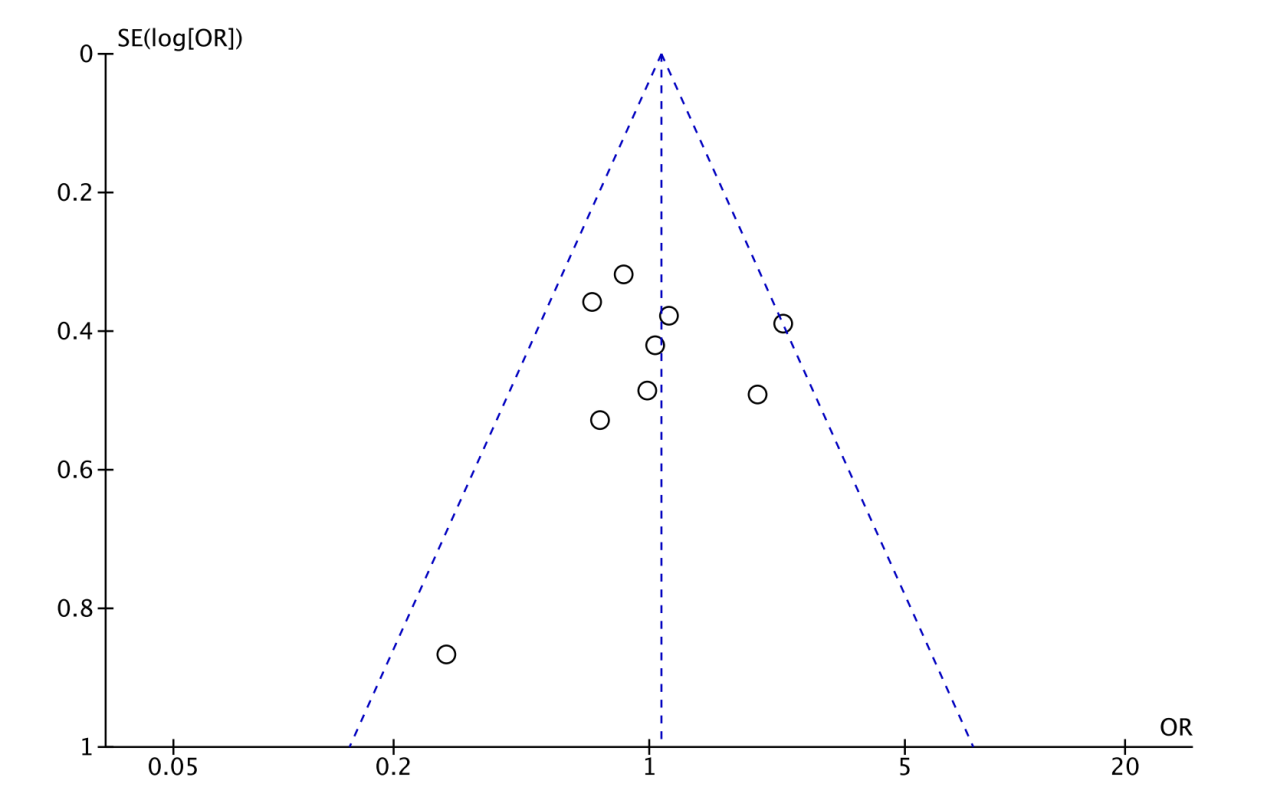


Figure 10.


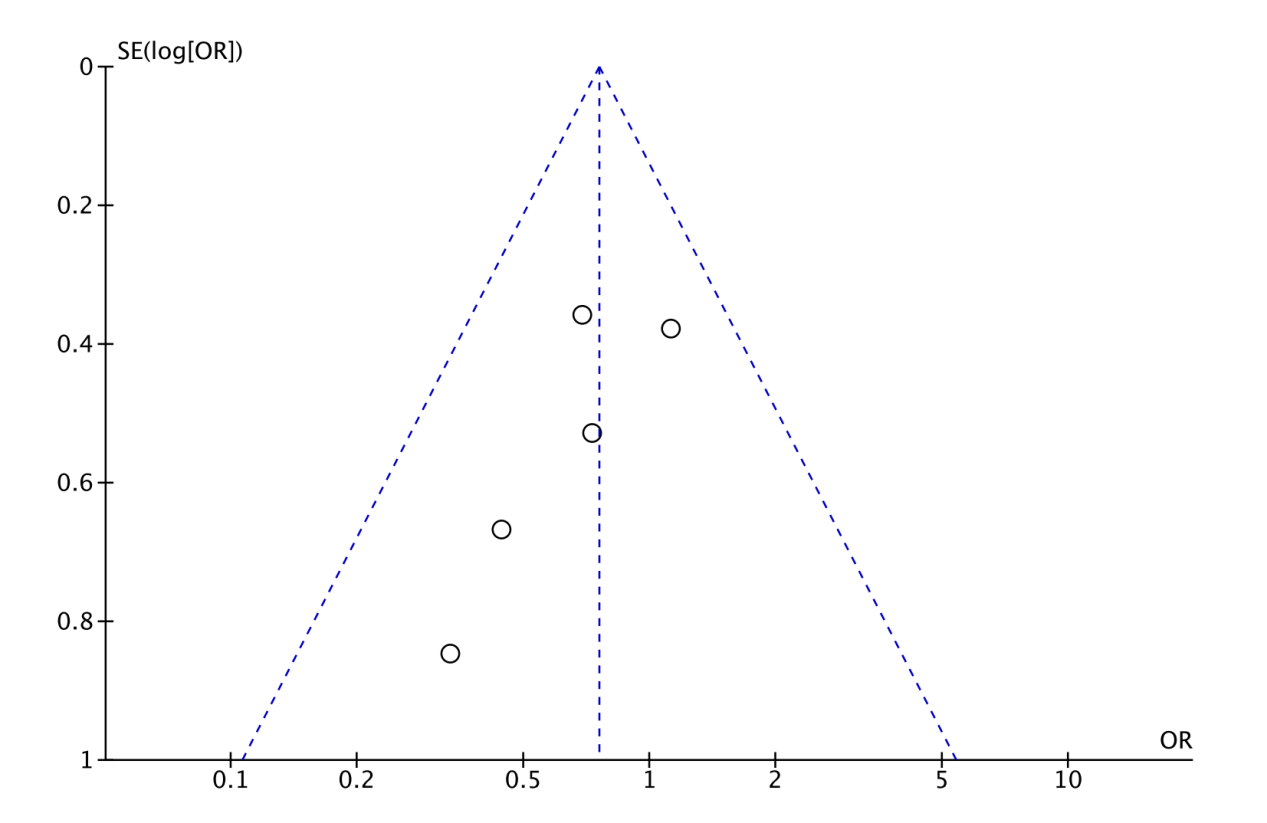


Figure 11.


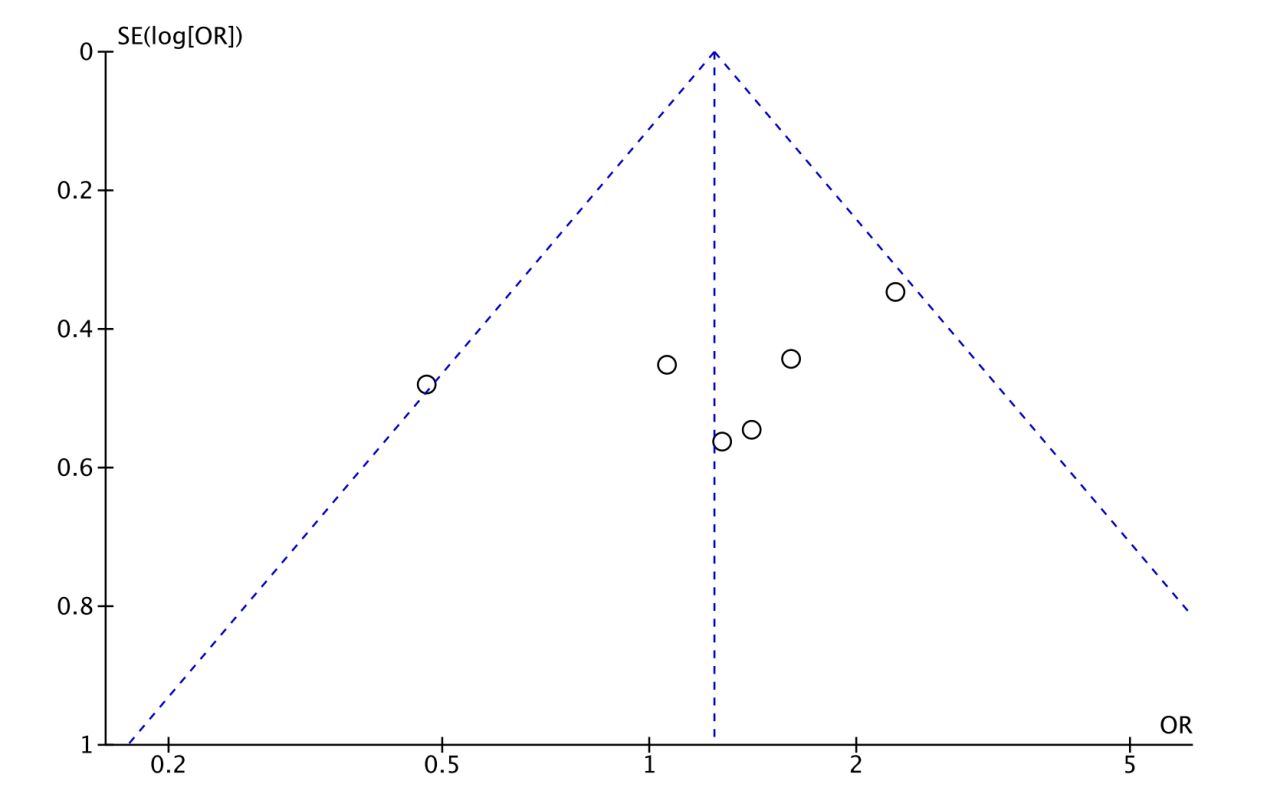


Figure 12.


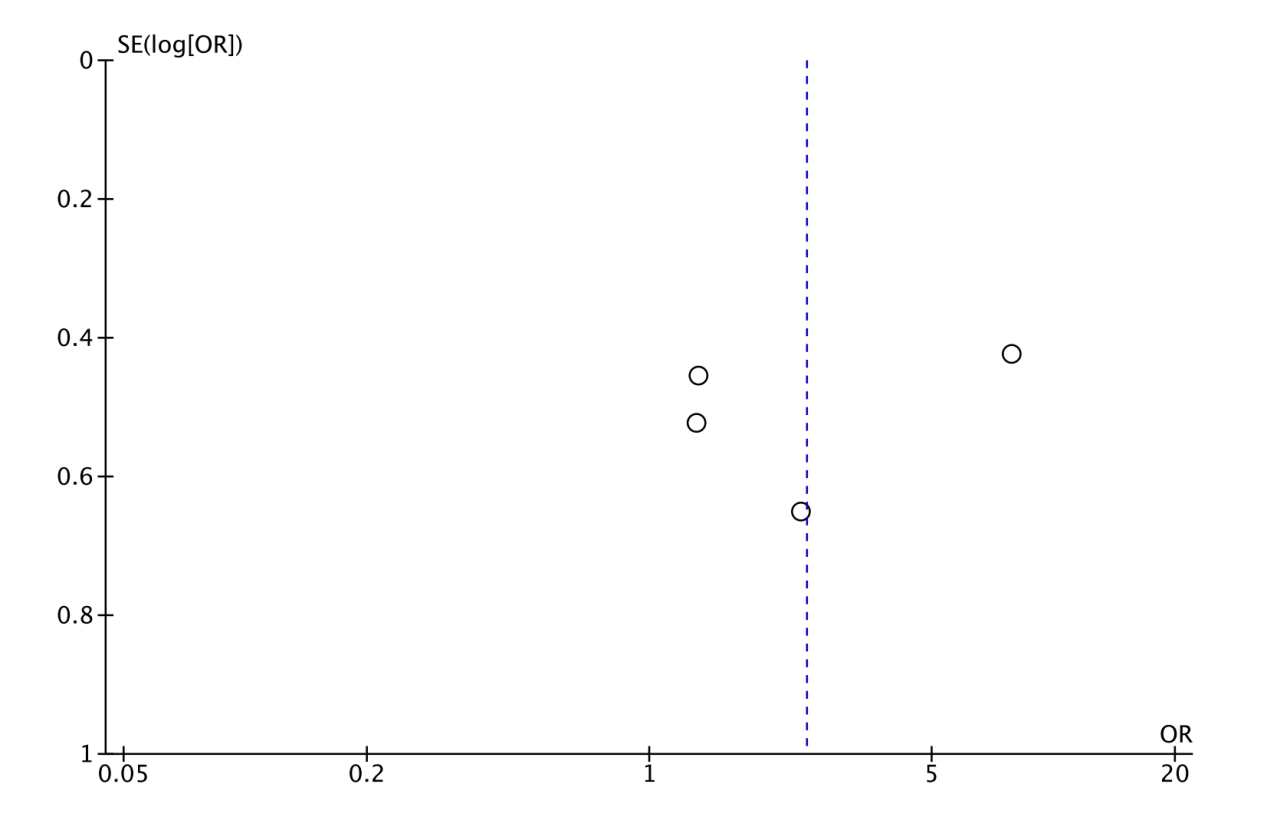


Figure 13.


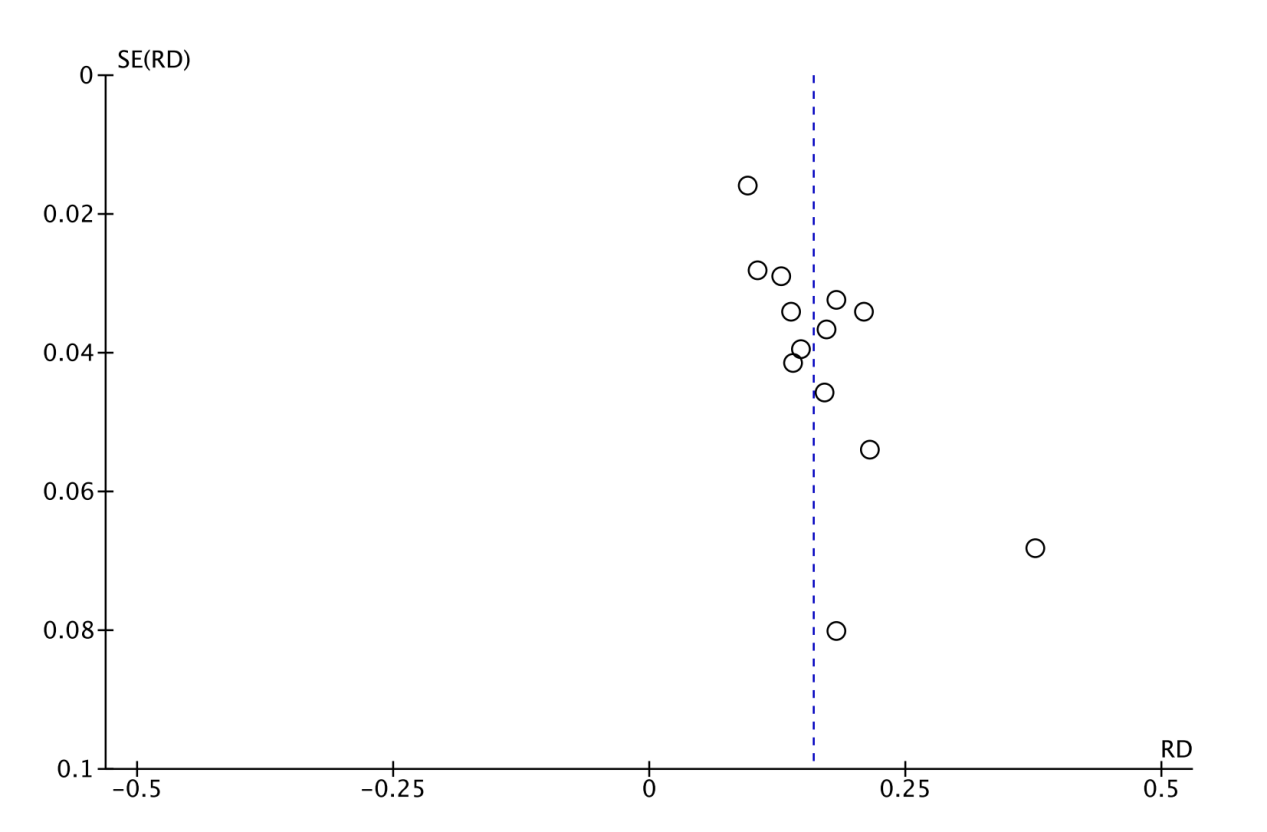

Supplement: Supplementary file 1 — Supplementary Material 1 [file 12957_2024_3363_MOESM1_ESM.docx]
